# Evaluation of decision to delivery time interval and its effect on feto-maternal outcomes and associated factors in category-1 emergency caesarean section deliveries: prospective cohort study

**DOI:** 10.1186/s12884-020-2828-z

**Published:** 2020-03-17

**Authors:** Mamaru Mollalign Temesgen, Amare Hailekirose Gebregzi, Habtamu Getinet Kasahun, Seid Adem Ahmed, Yophtahe Berhe Woldegerima

**Affiliations:** Department of Anesthesia, College of Medicine and Health Science, University of Gondar, Gondar, Ethiopia

**Keywords:** Caesarean section, Decision to delivery interval, Feto-maternal outcome, Ethiopia

## Abstract

**Background:**

Category-1 emergency caesarean section delivery is the commonly performed surgical procedure in pregnant women associated with significant mortality and morbidity both in the mother and fetus. The decision to delivery time interval is recommended to be less than 30 min by the Royal College of Obstetricians and Gynecologists as well as the American College of Obstetricians and Gynecologists. This study was designed to evaluate the decision to delivery time interval and its effect on feto-maternal outcomes and the associated factors during category-1 emergency caesarean section deliveries.

**Method:**

A prospective observational cohort study was conducted from March to May 2018 at the University of Gondar Comprehensive Specialized Hospital obstetrics Operation Theater and postnatal ward. A total of 163 clients who were undergone category-1 emergency caesarean section were included in this study. Statistical analysis was performed using SPSS version 20 (IBM Corporate). Bivariate and multivariate logistic regression with a 95% confidence interval was used to determine the association of decision to delivery time interval with predictor variables and feto-maternal outcomes.

**Results:**

Only 19.6% of women had a decision to delivery time interval below 30 min. The average decision to delivery time interval was 42 ± 21.4 min, the average time from the decision of category-1 emergency caesarean section arrival to the operation theater was 21.58 ± 19.76 min and from theater to delivery of anesthesia was 11.5 ± 3.6 min. Factors that were associated with prolonged decision to delivery time interval were: time taken to collect surgical materials (AOR = 13.76, CI = 1.12–168.7), time taken from decision and arrival to the operation theater (AOR = 0.75, CI = 0.17–3.25) and time taken from arrival at the operation theater to the immediate start of skin incision (AOR = 0.43, CI = 0.28–0.65).

**Conclusion:**

Delivery was not achieved within the recommended time interval in the majority of category-1 emergency caesarean sections. The average decision to delivery time interval was longer than the recommended time but it did not affect feto-maternal outcomes.

## Background

Category-1 emergency caesarean section delivery is one of the commonly performed surgical procedures in pregnant women which is associated with significant mortality and morbidity worldwide both in the mother and fetus [1, 2].

Ethiopia has made significant changes to improve feto-maternal health according to the World Health Organization report in 2015 [3]. But according to the Ethiopian Demographic Health Survey in 2011, Ethiopia has one of the highest maternal mortality ratio in Africa at 676 per 100,000 live births [4] and neonatal mortality rate of 35 per 1000 live births [5].

The national C/S rate in Ethiopia is about 2% but it varies widely among administrative regions due to unequal access [6].The rate increases worldwide beyond the recommended level of 10% and reached 30% [7].

Category 1 emergency C/Sis performed when there is an immediate threat to life (mother and fetus) and need of delivery within 30 min [2]. Avoiding the adverse neonatal effects of perinatal asphyxia has been one of the common indications for C/S deliveries in current obstetric practice [8].

Antenatal care (ANC) and good communication among health care providers are vital for better maternal and fetal outcome during category-1emergency C/S delivery [9].

When category-1 emergency C/S is indicated, the most important predictor for fetal and maternal outcome is decision to delivery interval (DDI), which is recommended to be within 30 min [10].

A retrospective cohort study done in Ghana showed that increased DDI is associated with adverse perinatal outcome [10]. The rates of maternal and neonatal complications were high in both extremes of maternal age [11].

The general objective of this study was to evaluate decision to delivery time interval, its effect on feto-maternal outcomes and associated factors during category-1 emergency cesarean section delivery.

## Methods

### Study design and area

A prospective cohort study was conducted from March to May 2018 among women who underwent category-1 emergency C/S at Gondar University Specialized Hospital obstetrics operation room, recovery room and wards located in Gondar town, Northwest Ethiopia.

### Source and study population

#### Source population

All pregnant mothers who underwent C/S at the University of Gondar Comprehensive Specialized Hospital (UOCGS).

#### Study population

Clients who underwent category-1 emergency cesarean section under both general and regional anesthesia at GUCSH during the study period.

### Inclusion and exclusion criteria

#### Inclusion criteria

All clients who underwent category-1 emergency C/S delivery under both general and regional anesthesia were included.

#### Exclusion criteria

All clients who underwent category-1 emergency C/S with preterm fetus, uterine rapture before decision, refused to give consent and fetus with gross congenital anomaly were excluded.

### Variables of the study

#### Dependent variables

Time of decision to delivery interval (DDI) and feto-maternal outcomes were dependent variables.

#### Independent variable

Independent variables were socio-demographic factors(Age, weight, height, BMI, gestational age, ANC follow up, educational level, number of previous C/S), experience of the obstetrician, duration of surgery, experience of the anesthetist, duration of anesthesia, ANC follow up, hemodynamic status of the client, availability of surgical materials, client information (did the client have knowledge about the complications due to prolonged DDI), team communication (early information delivery among the surgical team), availability of surgical team, unplanned conversion to general anesthesia, and availability of operating tables.

### Operational definition

- **Category-1 emergency caesarean section**: immediate threat to the life of the woman or fetus which needs delivery of the fetus within 30 min [12].
- **Transfer time**: the time taken from decision for C/S to arrival in the operation theater [13].
- **Anesthesia time**: the time taken from transfer and immediate start of anesthesia to skin incision [13].
- **Operation time**: the time taken from skin incision to delivery of the fetus [14].
- **DDI**: The time from decision of C/S to fetal delivery [15].
- **Peri-natal outcome**: neonatal mortality and morbidity or birth without complications [16].
- **Fever**: the American College of Critical Care Medicine, the International Statistical Classification of Diseases, and the Infectious Diseases Society of America define fever as a core temperature of 38.3 °C or higher [17].
- **Sever pre-eclampsia**: systolic blood pressure of 160 mmHg or higher or diastolic blood pressure of 110 mmHg or higher in two occasions at least 4 h apart [18].
- **Eclampsia**: the occurrence of new onset of seizure in a mother with pre-eclampsia [18].
- **Severe APH**: an acute blood loss > 1500 ml with cold clammy skin, tachycardia, tachypnea and hypotension [19].

### Sample size and sampling procedure

#### Sample size determination

The sample size was estimated by taking the achievement of DDI below 30 min (12.3%)among emergency C/S deliveries from a study done in Tanzania with the assumptions of single population proportion at 5% margin of error, and at 95% of confidence interval [20]. So, it was calculated as:

$$ n=\frac{{\left(Z\raisebox{1ex}{$\alpha $}\!\left/ \!\raisebox{-1ex}{$2$}\right.\right)}^2\rho \left(1-\rho \right)}{\varepsilon^2} $$

$$ n=\frac{(1.96)^2\times 0.123\left(1-0.123\right)}{(0.05)^2}=\mathbf{166} $$

A total of **166** participants were required.

#### Sampling procedure

Every consecutive women who underwent category-1 emergency C/S under both general and spinal anesthesia during the study period was included.

#### Data collection procedures

Data was collected by using a structured questionnaire. Socio-demographic variables, the time of decision of C/S, indication of C/S, time of OT transfer, time taken to deliver anesthesia, the total time taken from decision to delivery of the fetus and the time of anesthesia team informed were collected from patients’ chart and direct observation.

The time of decision for category-1 emergency C/S was recorded at the time the obstetrician decided to do caesarean section. Subsequently the time of transfer to the operation theater, type of anesthesia, time taken for administration of anesthesia and tame taken for operation were also recorded.

The pre-operative and post-operative maternal vital signs (BP, PR, temperature, spo2 and urine output) and BMI were recorded. Post-operatively, mothers were evaluated for short term maternal outcomes (fever, wound infection, bladder injury, hysterectomy, need for blood transfusion, administration of anti-convulsants, administrations of diuretics, blood loss and maternal death) until the 3^rd^post-operative day on which they would be discharged.

Neonatal outcomes were evaluated at the 1st and 5th minutes by using the Apgar score, need of intubation, cardiopulmonary resuscitation, need of admission to neonatal intensive care unit (NICU) and neonatal death.

#### Data quality control

Two BSc degree graduate anesthetists were selected and trained how to collect the data and supervised by the investigators. Pre-test was done on 5% of the sample to ensure the quality of the data and appropriate ammendments done. Data from pre-test was not included in the main study. The data was checked for completeness, accuracy, clarity and cleaned up y the principal investigator.

#### Data management and analysis

The data was coded, entered and analyzed using SPSS version 20 (IBM Corporate). Descriptive statistics was done. Categorical variables were presented in frequency and percentage. Continuous variables were presented in mean ± SD or median (IQR) according to results of Shapiro-Wilk normality test. Bivariate and multivariate binary logistic regression analyses were carried out to identify predictors. The strength of the association was assessed using odds ratio and 95% confidence intervals. A *p*-value less than 0.05 was considered as statistically significant. Finally, results were presented in tables and figures.

#### Ethical consideration

Ethical clearance was obtained from University of Gondar Collage of Medicine and Health Sciences, School of Medicine Ethical Review Committee. A written informed consent was taken from each study participant after detailed explanation. Every participant was allowed to discontinue participation if did not want to finish it. Also the participants were assured that their treatment and other benefits they can gain from the hospital will not be interrupted due to their withdrawal. Participants who had complication were given advice, and their respective physicians and midwives were alerted about the problem. Confidentiality was ensured by removing identifiers and locking the questionnaires in a secured area.

## Results

### Socio-demographic and clinical characteristics

A total of hundred and sixty-six clients were enrolled in the current study with a response rate of 98.2%and 3 of participants were excluded from the study because of incomplete data. Majority of participant’s 68 (41.7%) age was in the range of 25–29 years. Around 32(19.6%) of women had history previous C/S and almost a half of clients had college or university level of education. The mean weight, height and gestational age of participants were 66 ± 10 Kg, 1.61 ± 0.06 m and 38 ± 1 weeks, respectively. The majority of participants 85(52.1%) had BMI between 25 and 29.9 Kg/m^2^.The mean weight of the new born was 3.04 ± 0.19 Kg (Table 1). Most of clients were operated under spinal anesthesia. Single attempt lumbar puncture was demonstrated in 54(33.15%) participants and two, three, and four or more attempts were noted in 68(41.75%), 23(14.1%), and 2(1.2%) participants respectively.

**Table 1:** Feto-maternal socio-demographic characteristics of category-1 emergency caesarean section at Gondar University specialized Hospital, Northwest Ethiopia, March to May 2018 (*N* = 163)

Maternal characteristics	n (%)
age (years)	
< 20	7(4.3)
20–24	51(31.3)
25–29	68(41.7)
30–34	25(15.3)
35–39	11(6.7)
≥ 40	1(0.6)
Number of previous C/S	
0	131(80.4)
1	14(8.6)
2	16(9.8)
≥ 3	2(1.2)
Educational level	
No formal education	28(17.2)
Primary school	42(25.8)
Secondary school	46(28.2)
Collage/university	47(28.8)
Mean ± SD weight (kg)	66± (10)
Mean ± SD height (meter)	1.61 ± (0.06)
Mean ± SD gestational age (weeks)	38± (1)
Fetal mean ± SD weight (kg)	3.04 ± (0.19)

*SD* standard deviation

The mean ± SD preoperative systolic blood pressure, diastolic blood pressure, pulse rate, respiratory rate, oxygen saturation, temperature and urine output were 125 ± 16 mmHg, 73.5 ± 10.9 mmHg, 97.8 ± 14.9 beats/minute, 20 ± 2.5 breaths/minute, 96 ± 2%, 36 ± 04°c and 55 ± 50.8 ml respectively. The mean ± SD post-operative systolic blood pressure, diastolic blood pressure, pulse rate, respiratory rate, oxygen saturation, temperature and urine output were 106.5 ± 16.7 mmHg, 62.65 ± 9.96 mmHg, 90 ± 11.08 beats/minute, 19 ± 1.9 breaths/minute, 96.4 ± 1.9%, 35.9 ± 9.8 °C and 192 ± 95.8 ml respectively. None of the vital signs was associated with DDI or feto-maternal outcomes.

**Fig. 1:**
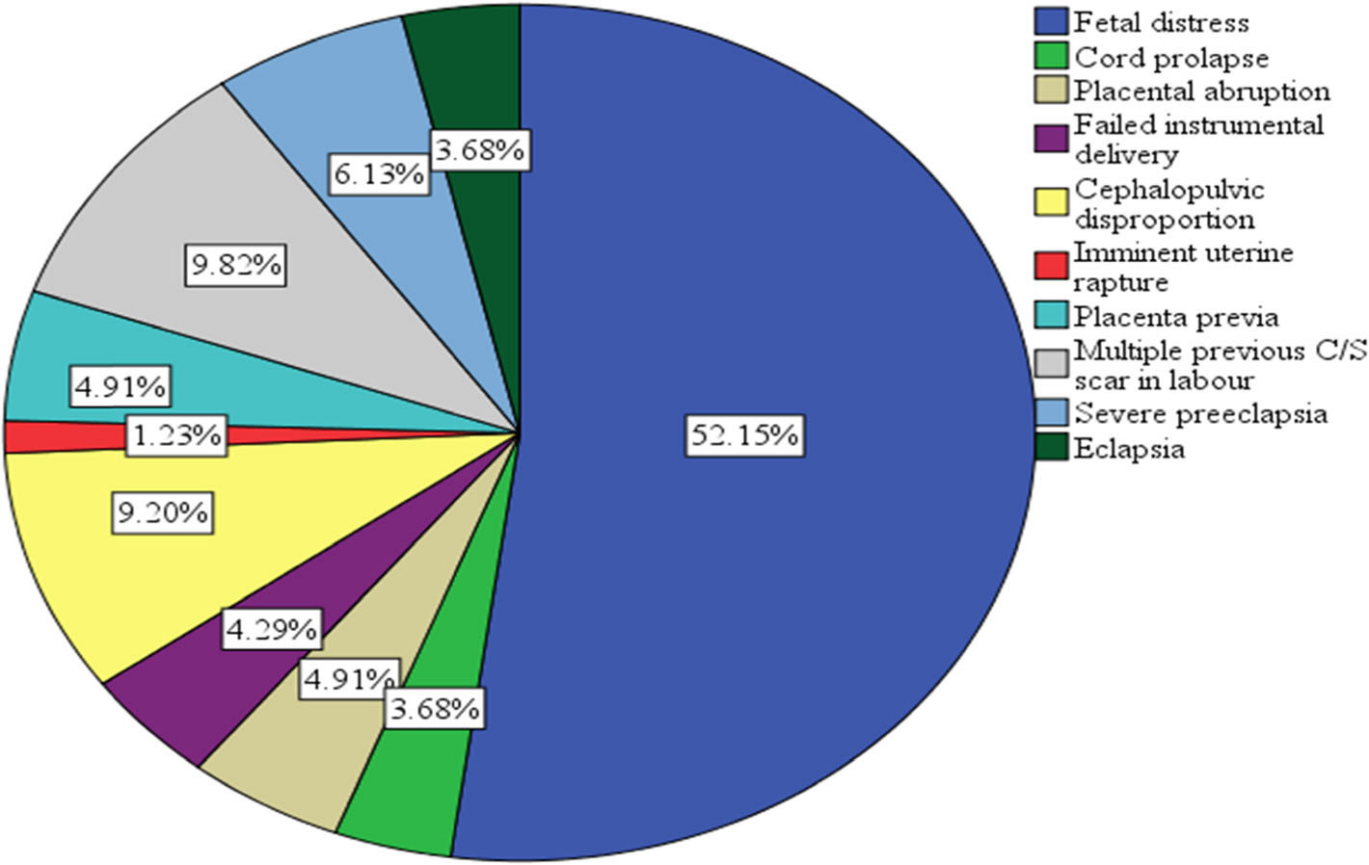
A pie chart shows indications of category-1 emergency caesarean section in Gondar University specialized Hospital Northwest Ethiopia, March to May 2018(percentage) (*N* = 163)

The leading indications for category-1 emergency C/S were fetal distress (52.15%), multiple previous C/S scars with ongoing labor (9.82%) and cephalopelvic disproportionate (9.20%). Others are prolapsed umbilical cord, placental abruption, failed instrumental delivery, imminent uterine rupture, placenta previa, severe pre-eclampsia and eclampsia (Fig. 1). Around 5.5% of clients had combination of indications.

### Decision to delivery time interval, and other intervals

The recommended decision to delivery time interval (DDI below 30 min) was achieved only in 32 (19.6%) of category-1 emergency C/S. The mean ± SD of DDI was 42 ± 21.4 min. The mean ± SD of time taken from decision of C/S to transfer to OT was 21.58 ± 19.76 min and from arrival at the operation theater to skin incision was 11.5 ± 3.6 min (Table 2).

**Table 2:** Decision to delivery time intervals, duration of surgery and anesthesia of category-1 emergency caesarean section at Gondar University specialized Hospital Northwest Ethiopia, March to May 2018(*N* = 163)

Characters	Values
Time taken from decision to theater (minute)	21(±19.76)*
Time taken for delivering anesthesia (minute)	11.5(±3.6)*
Decision to delivery time interval > 30 min	131(80.4)**
Decision to delivery time interval ≤ 30 min	32(19.6)**
Decision to delivery time interval (minute)	42(±21.4)*
Duration of anesthesia (minute)	55(50–65)***
Duration of surgery (minute)	49(43–58)***

*SD* standard deviation, *IQR* interquartile range (maen ± SD)*,n(%)** and madian(IQR)***

### Predictors of decision to delivery time interval in category-1 emergency C/S

Around 152 (93.3%) of clients had ANC follow up and out of those, 31 (20.4%) had DDI below 30 min. The majority of cases were operated by senior residents and out of those, 26 (20.8%) had DDI below 30 min. Most of clients 63 (82.9%) were anesthetized by BSc holder anesthetists and out of those, 13 (17.1%) had DDI below 30 min (Table 3).

**Table 3:** Bivariate logstic regression analyses results: Health professionals and clients related factors that can delay decision to delivery time interval in category-1 emergency caesarean section, at Gondar University specialized Hospital Northwest Ethiopia, March to May 2018(x-tab and OR with 95% CI)((*N* = 163)

Variables	Decision to delivery time interval		OR
	> 30 min n (%)	≤30 min n (%)	COR (95%CI)
BMI (kg/m2)			
18.5–24.9	59(85.5)	10(14.5)	1
25–29.9	65(76.5)	20(23.5)	1.81(0.79–4.19)
≥ 30	7(77.8)	2(22.2)	1.69(0.31–9.31)
Number of previous C/S			
0	102(77.9)	29(22.1)	1
1	14(100)	0(0)	0.28(0.02–4.69)
2	14(87.5)	2(12.5)	0.5(0.11–2.32)
≥ 3	1(50)	1(50)	3.52(0.21–57.97)
ANC follow up			
Yes	121(79.6)	31(20.4)	1
No	10(90.9)	1(9.1)	0.39(0.05–3.17)
Educational level			
No formal education	23(82.1)	5(17.9)	1
Primary school	33(78.6)	9(21.4)	1.25(0.37–4.230)
Secondary school	36(78.3)	10(21.7)	1.28(0.39–4.22)
Collage/university	39(83)	8(17)	0.94(0.28–3.23)
Experience of surgeons			
Junior residents	32(84.2)	6(15.8)	1
Senior residents	99(79.2)	26(20.8)	1.4(0.53–3.71)
Experience of anesthetist			
BSC students	12(80)	3(20)	1
BSC holders	63(82.9)	13(17.1)	0.7(0.12–3.56)
MSC student	42(79.2)	11(20.8)	0.58(0.178–1.89)
MSC holders	14(73.7)	5(26.3)	0.73(0.17–2.48)

*C/S* caesarean section, *ANC* antenatal care, *BMI* Body mass index

Time elapsed to collect materials, time of decision of C/S, time taken from decision to the operation theater, time taken to deliver anesthesia and operation time were associated with prolonged DDI (Table 4).

**Table 4:** Bivriate and multivariate logstic regression analyses results: Predictors of decision to delivery time interval in category-1 emergency caesarean section, Gondar University specialized Hospital Northwest Ethiopia, March to May 2018 (X-tab and OR with 95%CI) (*N* = 163)

Reason for delay	Decision to delivery time interval		OR	
	> 30 min n (%)	≤30 min n (%)	COR (95%CI)	AOR (95%CI)
Availability of materials				
Easily available	101(77.1)	30(22.9)	1	1
Tooke time to collect	30(93.8)	2(6.2)	4.46(1.01–19.73)*	13.76(1.12–168.7)*
Time of decision				
Day	87(86.1)	14(13.9)	1	1
Night	44(71)	18(29)	0.39(0.18–0.86)*	0.73(0.17–3.25)
Type of anesthesia				
Reginal	121(82.3)	26(17.7)	1	
General	10(62.5)	6(37.5)	0.36(0.12–1.07)	
Skin incision to delivery				
≤ 5 min	7(50)	7(50)	1	1
> 5 min	124(83.2)	25(16.8)	0.20(0.07–0.63)*	0.18(0.02–2.03)

* P-value < 0.05

Other factors such as unavailability of clinicians (anesthetists 8(4.9%), surgeons 3(1.8%), and midwifes 3(1.8%)), hesitation of the client to give consent 18(11%), unplanned conversion to general anesthesia 2(1.2%), lack of operation table 11(6.7%), difficulty of intravenous access 7(4.3%), waiting for CBC results 1(0.6%), waiting of senior surgeon 1(0.6%) and waiting for consultant anesthetist 1(0.6%) had no association with prolonged DDI .

### Fetal outcomes in category-1emergency C/S

**Fig. 2:**
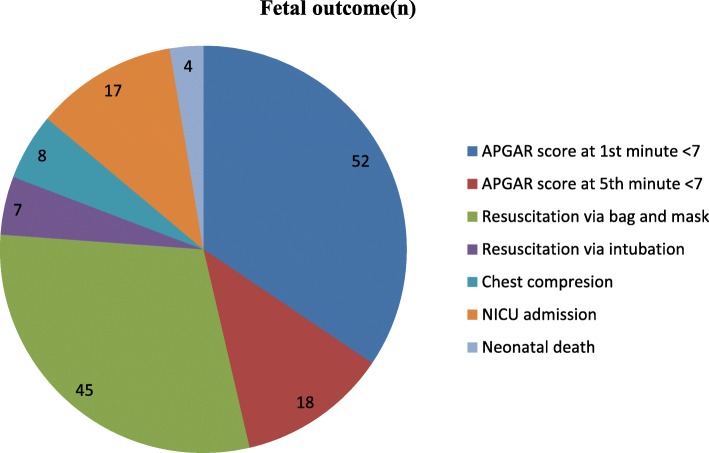
A pie chart shows fetal outcomes of category-1 emergency caesarean section in Gondar University specialized Hospital Northwest Ethiopia, March to May 2018(percentage) (*N* = 163)

A total of 163 deliveries were included in the study. Apgar score < 7 was recorded in 52 newborns at the 1st minute and in18 at the 5th minute. Bag and mask resuscitation was done in 45 newborns, 7 newborns were intubated, chest compression was done for 7 newborns and 17 were admitted to the neonatal intensive care unit and 4 newborns died (3 still birth and 1 after delivery) (Fig. 2). Among 131 newborns who delivered with a DDI longer than 30 min, 40 had Apgar score < 7 at the first minute, 13 had Apgar score < 7 at the 5^th^minute, 38 had resuscitation via bag-mask ventilation, 5 had intubation, and 6 had chest compression (Table 5).

**Table 5:** Bivariate logstic regression analyses results: Fetal outcome in category-1emergency caesarean section, at Gondar University specialized Hospital Northwest Ethiopia, March to May 2018(X-tab and OR with 95%CI) (*N* = 163)

Variable	Neonatal outcomes		OR
	APGAR score at 1^**st**^ minute		COR (95%CI)
Decision to delivery time interval	**< 7 n (%)**	**≥7 n (%)**	
> 30 min	**40(30.5)**	**91(69.5)**	**1**
≤30 min	12(37.5)	20(62.5)	1.37 (0.61–3.06)
	**APGAR score at 5**^**th**^**minute**		
	< 7 n (%))	**≥7 n (%)**	
> 30 min	13 (9.9)	**118 (90.1)**	**1**
≤30 min	5 (15.6)	27 (84.4)	1.68 (0.55–5.12)
	**Bag mask resuscitation**		
	Yes n (%)	No n (%)	
> 30 min	38 (29)	93 (71)	1
≤30 min	**7 (21.9)**	**25 (78.1)**	0.69(0.29–1.72)
	**Resuscitation via intubation**		
	Yes n (%)	No n (%)	
> 30 min	**5 (3.8)**	**126 (96.2)**	**1**
≤30 min	2(6.2)	30(93.8)	1.68 (0.31–9.08)
	**Chest compression**		
	Yes n (%)	No n (%)	
> 30 min	6 (4.6)	125 (95.4)	1
≤30 min	2(6.2)	30(93.8)	1.39(0.27–7.23)
	**NICU admission**		
	Yes n (%)	No n (%)	
> 30 min	15 (11.5)	116 (88.5)	1
≤30 min	2(6.2)	30(93.8)	0.52(0.11–2.38)
	**Neonatal death**		
	Yes n (%)	No n (%)	
> 30 min	3 (2.3)	128 (97.7)	1
≤30 min	1 (3.1)	31 (96.9)	1.38 (0.14–13.69)

*Apgar* appearance, pulse, grimace, activity, respiration

### Maternal outcome in category-1emergency C/S

**Fig. 3:**
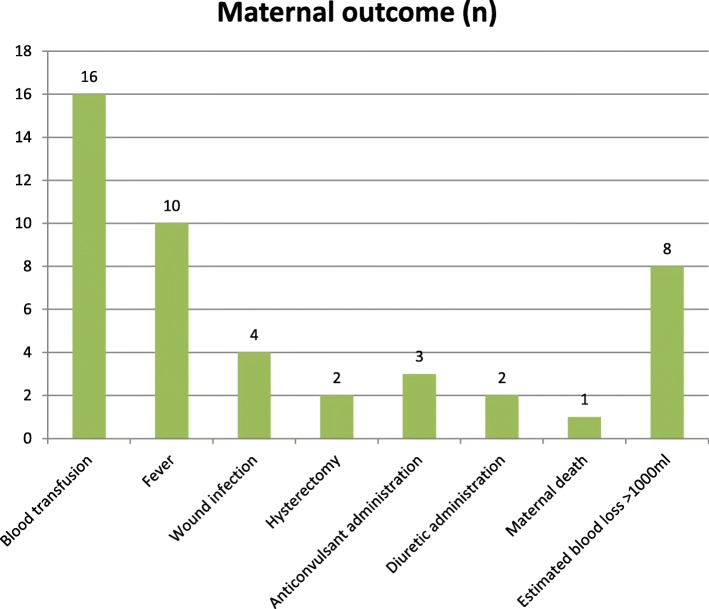
A bar graph shows maternal outcomes of category-1 emergency caesarean section in Gondar University specialized Hospital Northwest Ethiopia, March to May 2018(frequency) (*N* = 163)

Out of 163 mothers who delivered with category-1 emergency C/S, 16 were transfused, 10 had developed fever, 4 had wound infection, 2 had hysterectomy, 8 had lost blood which was estimated to be more than 1000 ml and 1 had died (Fig. 3). Among 32 mothers whose DDI was longer than 30 min, 12 were transfused, 8 had developed fever, 2 had developed wound infection and 1 had hysterectomy (Table 6).

**Table 6:** Bivariate logstic analyses results: Maternal outcome in category-1 emergency caesarean section, at Gondar University specialized Hospital Northwest Ethiopia, March to May (X-tab and OR with 95%CI) (*N* = 163)

Variable	Maternal outcome		OR
	Blood transfusion		
**Decision to delivery time interval**	**Yes n (%)**	**No n (%)**	**COR (95%CI)**
**> 30 min**	12 (9.2)	119 (90.8)	**1**
**≤30 min**	4 (12.5)	28 (87.5)	1.42 (0.43–4.72)
	**Fever**		
	Yes n (%)	No n (%)	
**> 30 min**	8 (6.1)	123 (93.9)	1
**≤30 min**	2(6.2)	30(93.9)	1.03 (0.21–5.08)
	**Wound infection**		
	Yes n (%)	No n (%)	
**> 30 min**	2(1.5)	129 (98.5)	1
**≤30 min**	2(6.2)	30(93.8)	4.3 (0.58–31.77)
	**Hysterectomy**		
	Yes n (%)	No n (%)	
**> 30 min**	1(0.8)	130 (99.2)	**1**
**≤30 min**	1 (3.1)	31 (96.9)	4.19 (0.26–68.9)
	**Blood loss**		
	> 1000 ml	< 1000 ml	
**> 30 min**	7 (5.3)	124 (94.7)	1
**≤30 min**	1 (3.1)	31 (96.9)	0.57 (0.07–4.82

### Discussion

Decision to delivery time interval is a time range between decision for C/S and delivery of a newborn. This is the critical time interval that determines the feto-maternal outcome in category-1 emergency C/S [8]. The current study showed that only 19.6% of women who underwent category-1 emergency C/S were delivered within the recommended DDI of 30 min. The mean ± SD of DDI was 42 ± 21.4 min which is similar with a study done by Sunanda et al. in which the mean ± SD was 36.3 ± 17 min [21]. The difference might be due to sample size. Another study done in Oman concluded that a DDI below 30 min was achieved in 23.8% of category-1 emergency C/S [22] which is comparable with the current study. On the other hand, a study done in Benin teaching hospital has showed that a DDI below 30 min was achieved in only 5.7% of emergency C/S and the mean ± SD of DDI was 106.3 ± 79.5 min [23]. This finding is low in comparison with the current study.

In this study, time to collect surgical materials had a positive association with prolonged DDI in category-1 emergency C/S(AOR =, CI =, p=).This is consistent with a research done by Tak Yeung Leung et al. which has stated that a DDI below 30 min was achievable if the operation facilities were easily available [24]. Another study has showed that lack of surgical equipments was the main factor for prolonged DDI time [25]. A DDI below 30 min was difficult to attain in emergency C/S due to the infrastructural challenges [23].

In the current study, the mean time from decision to arrival to the operation theater was 21.58 ± 19.76 min. A research done by Wong et al. concluded that the major determinant for prolonged DDI was the time taken for transfer of clients to the OT [26]. Another study also has stated that preparation and transfer to the operation theater have significantly prolonged DDI with the average of 15.9 min [27].The mean time taken to deliver anesthesia after the clients were arrived at the operation theater was 11.5 ± 3.6 min. A study has showed that delays in the preparation and administration of anesthesia were significantly associated with prolongation of DDI [28].

Category-1 emergency C/S done in the night time had generally shorter DDI when it compared with the day time which was 18(29%) vs 14(13.9%). This result was comparable with the study done in Nysamba Hospital, Uganda which has stated that C/S done during the day time had prolonged DDI than those done in the night [28]. This can be explained by; during the day time the operating tables might be occupied by elective cases.

A retrospective cohort study done in the University of Benin teaching hospital has showed that the most frequent causes for delay in emergency C/S were anesthetists delay and busy OT [29]. This finding is against with the current study and might be explained by committed anesthetists and availability of free operation tables in our setup.

The indication for more than a half of the category-1 emergency C/S was fetal distress. This was comparable with a study done by Zwuditu et al. [30] Most of the indications were also consistent with that of a research done by Dr. Ban Leong et al. [13, 28]. A DDI below 30 min was best achieved when the indication was failed instrumental delivery. Fifty-seven percent of mothers with the indication of failed instrumental delivery have delivered within 30 min during C/S. This might be due to the locations the delivery rooms which are very near to the OT and make easy to transfer the mother to the OT immediately. In contrast, another study has showed that DDI below 30 min was achieved among women with the indication of non–reassuring fetal heart rate. The 3/4th of women with this indication has delivered within the recommended DDI [31].

The most of participants (90.2%) were operated under spinal anesthesia. This result was in accordance with a previous study in which 97.2% of category-1 emergency C/S were done under spinal anesthesia [23]. General anesthesia was administered in 16 clients and the attainments of DDI below 30 min was 37.5% [26]. A prospective study done by Mackenzie IZ et al. claimed that general anesthesia was significantly associated with shorter DDI than regional anesthesia for emergency caesarean section [32].

Most category-1 emergency C/S (76.7%) in our study was performed by senior residents (R3 and R4) and 23.3% by junior resident (R2). Achievement of DDI below 30 min was 20.8 vs 15.8% respectively. Senior obstetricians were not evolved in any of cases. However, the experience of obstetric residents was not a statistically significant determinant of DDI. This is supported by a prospective study done by Mackenzie IZ et al. which stated that the seniority of the surgeon didn’t influence the DDI [32]. In the majority of the cases (44.6%), anesthetic care was provided by BSc degree graduate anesthetists and the remaining 32.5 and 11.7% by MSc degree in anesthesia students and MSc graduates respectively. The study has denied that experience of the anesthetists had no association with DDI. In contrast to our finding, there is a study that showed lack of experience of the obstetricians, anesthetists and hesitation of the pregnant mother to give consent can be barriers to achieve shorter DDI [25].

In this study there was no association between DDI and feto-maternal outcome. Most of the literature stated that, there was no association between DDI and feto-maternal outcome during category-1 emergency C/S [13, 28, 31]. On the other hand Jane Thomas et al. stated that only delays in DDI longer than 75 min were significantly associated with worse feto-maternal outcome in category-1 emergency C/S [33]. Despite these, another study has showed that during life threatening conditions, quicker delivery can result in better feto-maternal outcomes [24].

Many newborns with adverse outcome had DDI longer than 30 min but it was not statistically significant. This result is supported by previous studies that claimed longer DDI was not significantly associated with worse neonatal outcome [27, 34]. In contrast, another study has stated that there was significant improvement in feto-maternal outcome when DDI was below 20 min [35].

Among newborns who were delivered with DDI longer than 30 min, 40 had Apgar score < 7 at the 1^st^minute, 13 had Apgar score < 7 at the 5thminute, 38 had resuscitation via bag-mask ventilation, 5 had intubation, 6 had chest compression, 3 had NICU admission and 3 had died. On the other hand, when DDI was below 30 min, 12 had Apgar score < 7 at 1stminute, 5 had Apgar score < 7 at fifth 5th minute, 7 had resuscitation via bag-mask ventilation, 2 had intubation, 2 had chest compression, 2 had NICU admission and 1 had died.

Among 131 mothers who delivered with DDI longer than 30 min, 12 had blood transfusion, 8 had developed fever, 2 had developed wound infections, 1 had hysterectomy and 7 had lost blood which was estimated to be more than 1000 ml. On the other hand, among 32 women who delivered with DDI below 30 min, 4 had blood transfusion, 2 had developed fever, 2 had developed wound infections, 1 had hysterectomy, and 1 had lost blood which was estimated to be more than 1000 ml. Despite our findings, a prospective study has showed that around 27% of women had one or more complications during caesarean section in which category-1 emergency C/S delivery significantly associated with these complications [36].

We strongly recommend that materials which are important for emergency C/S should be readily available in the supply room and obstetric pharmacy, anesthetic and surgical care for all category-1 emergency C/S should be provided by senior anesthetists, residents and consultants. Time of preparation and transfer to the OT should be reduced. It would be better if further study is done by incorporating late feto-maternal outcomes with larger sample size.

### Strengths and limitations of the study

The strengths of this study is that subjects were homogeneous (category-1 emergency C/S) which could provide representative data and since it was prospective study which could make it appropriate to identify factors.

This study has not evaluated late feto-maternal outcomes, immediate fetal outcome was assessed only with the Apgar score due to the unavailability of umbilical cord blood рН analysis in the hospital and even if larger sample size is needed, due to short study period we included only 163 samples and might reduce the power of the study. These are limitations of the study.

## Conclusions

Decision to delivery time interval in category-1 emergency C/S at UOGCSH was longer than the recommended interval of time. Only 19.6% of women were delivered within the recommended DDI below 30 min. Time taken to collect materials, time taken for client preparation and transfer to the Operation Theater and time taken to deliver anesthesia were associated with prolonged DDI. The techniques of anesthesia, experience of anesthetists and obstetric residents, availability of clinicians were not associated with DDI. Prolonged DDI had no association with feto-maternal outcomes.

## Data Availability

Data and materials used in this study are available and can be presented by the corresponding author upon reasonable request: the data supporting our findings is found in the department of anesthesia and critical care at the University of Gondar data set system.

## Abbreviations

ANC: Antenatal care
APGAR: Appearance, pulse, grimace, activity and respiration
APH: Ante partal hemorrhage
CS: Caesarean section
DDI: Decision to delivery interval
GA: General anesthesia
GUH: Gondar University Hospital
ICU: Intensive care unit
MNH: Maternal and newborn health
OT: Operation theater
SA: Spinal anesthesia
